# Supplementary material for: Implanting oxophilic metal in PtRu nanowires for hydrogen oxidation catalysis
Source: Nat Commun. 2024 Feb 6;15:1097. doi: 10.1038/s41467-024-45369-x (PMC10847104; doi:10.1038/s41467-024-45369-x)
Supplement: Supplementary file 1 — Supplementary Information [file 41467_2024_45369_MOESM1_ESM.pdf]

Supplementary Information

Implanting oxophilic metal in PtRu nanowires for hydrogen oxidation catalysis

Zhongliang Huang[1#], Shengnan Hu[1#], Mingzi Sun[2], Yong Xu[3*], Shangheng Liu[1], Renjie Ren[4], Lin Zhuang[4], Ting-Shan Chan[5], Zhiwei Hu[6], Tianyi Ding[1], Jing Zhou[7], Liangbin Liu[1], Mingmin Wang[1], Yu-Cheng Huang[8], Na Tian[1], Lingzheng Bu[9*], Bolong Huang[2*] and Xiaoqing Huang[1,10*]

[1]State Key Laboratory for Physical Chemistry of Solid Surfaces, College of Chemistry and Chemical Engineering, Xiamen University, Xiamen, 361005, China.

[2]Department of Applied Biology and Chemical Technology, The Hong Kong Polytechnic University, Hung Hom, Kowloon, Hong Kong SAR, 999077, China.

[3] Nano-X Vacuum Interconnected Nano-X Vacuum Interconnected Workstation, Suzhou Institute of Nano-Tech and Nano-Bionics (SINANO), Chinese Academy of Sciences (CAS), Suzhou, Jiangsu, 215123, China.

[4]College of Chemistry and Molecular Sciences, Hubei Key Lab of Electrochemical Power Sources, Wuhan University, Wuhan, 430072, China.

[5]National Synchrotron Radiation Research Center, 101 Hsin-Ann Road, Hsinchu, 30076, Taiwan.

[6]Max Planck Institute for Chemical Physics of Solids, Nothnitzer Strasse 40, Dresden, 01187, Germany.

[7]Shanghai Institute of Applied Physics, Chinese Academy of Sciences, Shanghai, 201800, China.

[8]Department of Electrophysics, National Yang Ming Chiao Tung University, Hsinchu, 30010, Taiwan.

[9]College of Energy, Xiamen University, Xiamen, 361102, China.

[10]Innovation Laboratory for Sciences and Technologies of Energy Materials of Fujian Province (IKKEM), Xiamen, 361005, China.

[*]E-mail: yongxu@gdut.edu.cn; lzbu@xmu.edu.cn; bhuang@polyu.edu.hk; hxq006@xmu.edu.cn

[#]These authors contributed equally to this work.

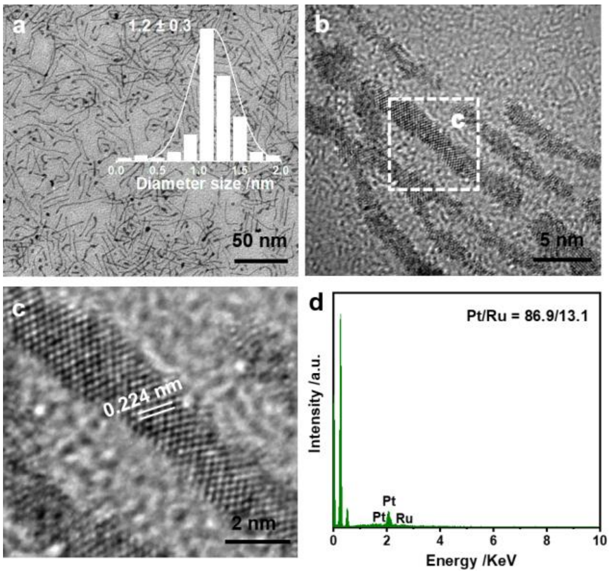

Supplementary Figure 1. TEM images and SEM-EDS profile. (a) TEM image, (b, c) HRTEM images, and (d) SEM-EDS profile of PR. Inset of (a) presents the diameter distribution of PR.

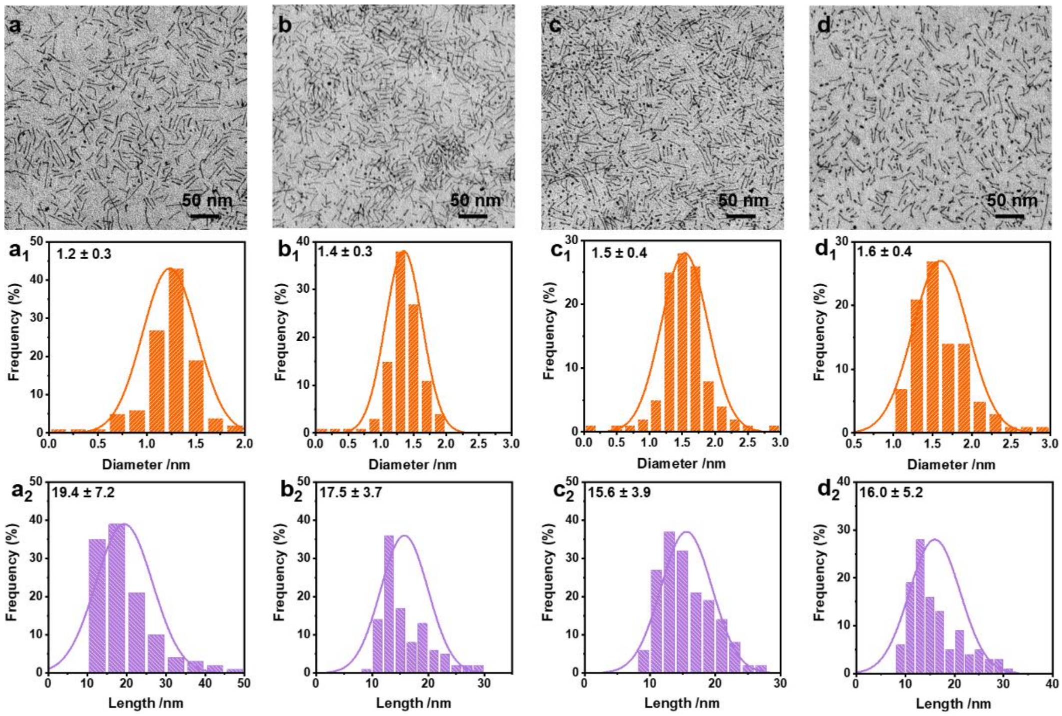

Supplementary Figure 2. TEM images and size distributions. TEM images of i-In$_x$-PR with different molar percentages of In: (a) 2%, (b) 3.9%, (c) 5.1%, and (d) 6.3%. (a_1, b_1, c_1, d_1) Diameter and (a_2, b_2, c_2, d_2) length distributions of (a_1, a_2) i-In$_2$-PR, (b_1, b_2) i-In$_{3.9}$-PR, (c_1, c_2) i-In$_{5.1}$-PR and (d_1, d_2) i-In$_{6.3}$-PR.

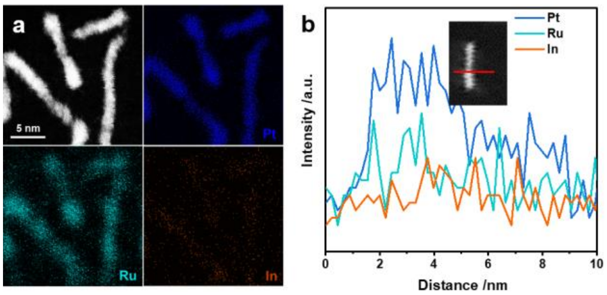

Supplementary Figure 3. TEM characterization. (a) HAADF-STEM image with elemental mappings and (b) TEM-EDS line-scanning elemental profile of i-In-PR.

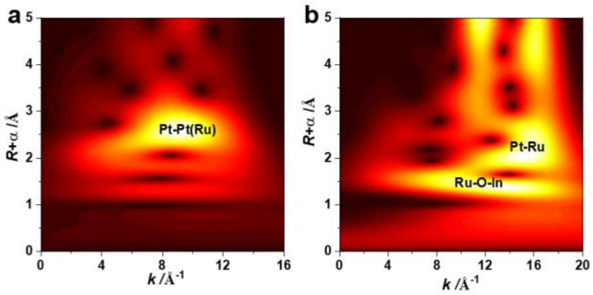

Supplementary Figure 4. Wavelet transformed. Wavelet transformed (a) Pt L_3-edge and (b) Ru K-edge EXAFS spectra of i-In-PR.

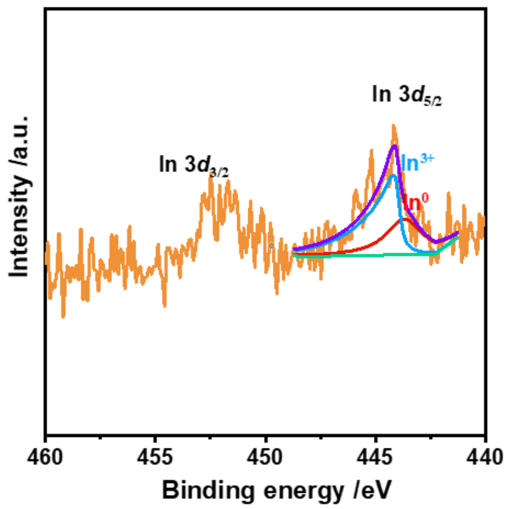

Supplementary Figure 5. XPS characterization. In 3*d* XPS spectrum of i-In-PR.

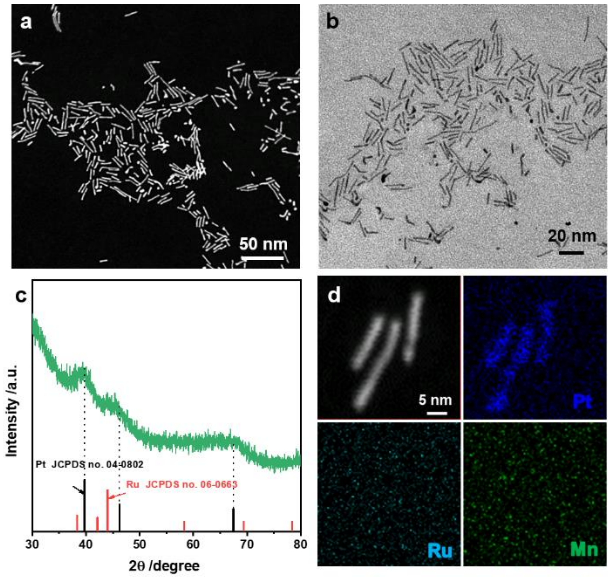

Supplementary Figure 6. Structural characterizations. (a) HAADF-STEM image, (b) TEM image, (c) XRD pattern, and (d) HAADF-STEM image with elemental mappings of i-Mn-PR.

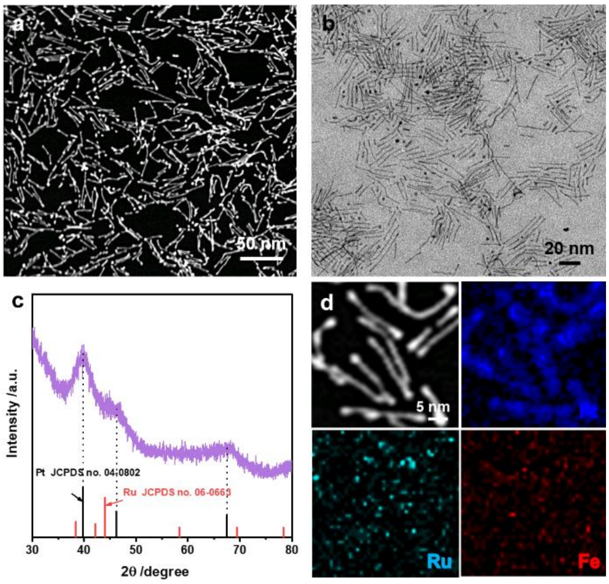

Supplementary Figure 7. Structural characterizations. (a) HAADF-STEM image, (b) TEM image, (c) XRD pattern, and (d) HAADF-STEM image with elemental mappings of i-Fe-PR.

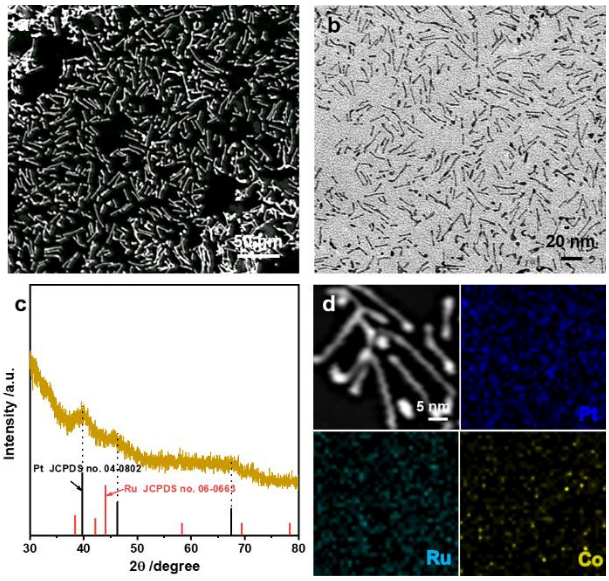

Supplementary Figure 8. Structural characterizations. (a) HAADF-STEM image, (b) TEM image, (c) XRD pattern, and (d) HAADF-STEM image with elemental mappings of i-Co-PR.

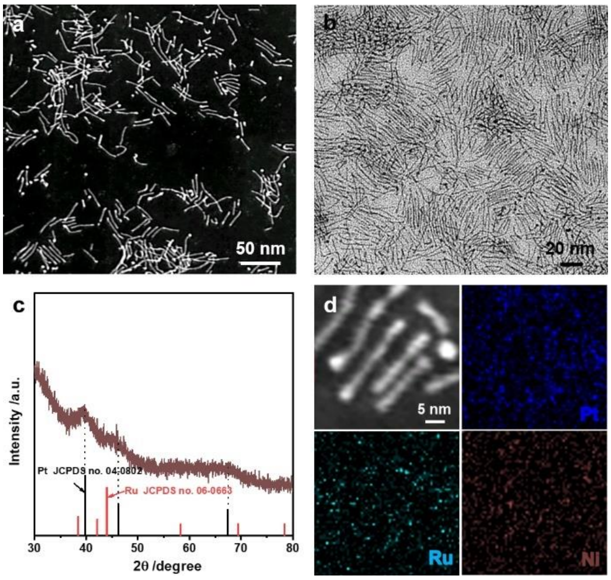

Supplementary Figure 9. Structural characterizations. (a) HAADF-STEM image, (b) TEM image, (c) XRD pattern, and (d) HAADF-STEM image with elemental mappings of i-Ni-PR.

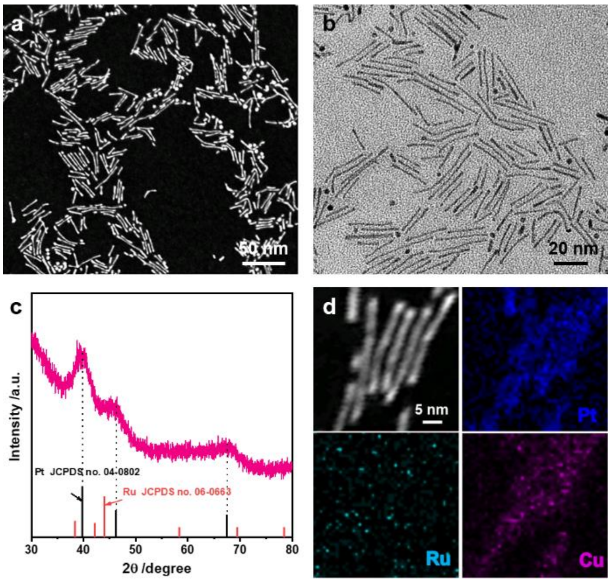

Supplementary Figure 10. Structural characterizations. (a) HAADF-STEM image, (b) TEM image, (c) XRD pattern, and (d) HAADF-STEM image with elemental mappings of i-Cu-PR.

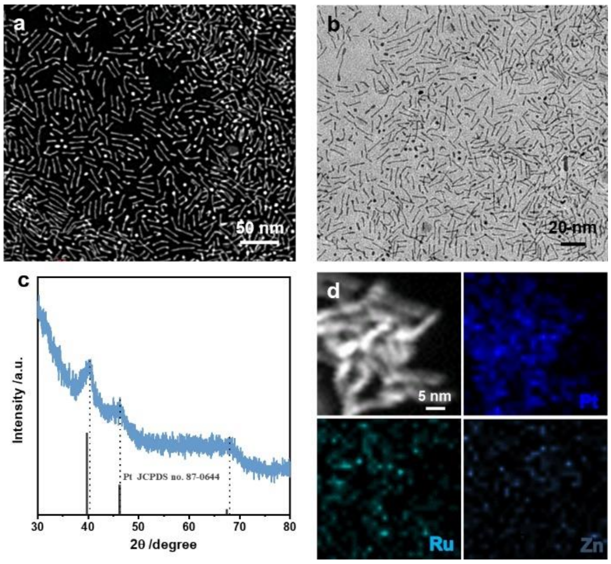

Supplementary Figure 11. Structural characterizations. (a) HAADF-STEM image, (b) TEM image, (c) XRD pattern, and (d) HAADF-STEM image with elemental mappings of i-Zn-PR.

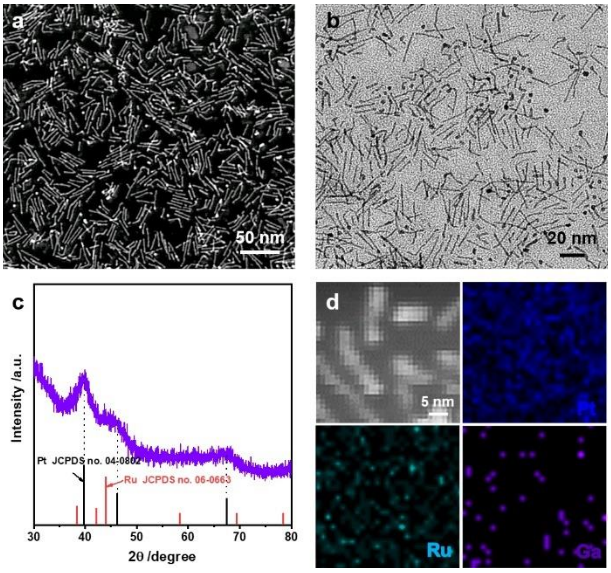

Supplementary Figure 12. Structural characterizations. (a) HAADF-STEM image, (b) TEM image, (c) XRD pattern, and (d) HAADF-STEM image with elemental mappings of i-Ga-PR.

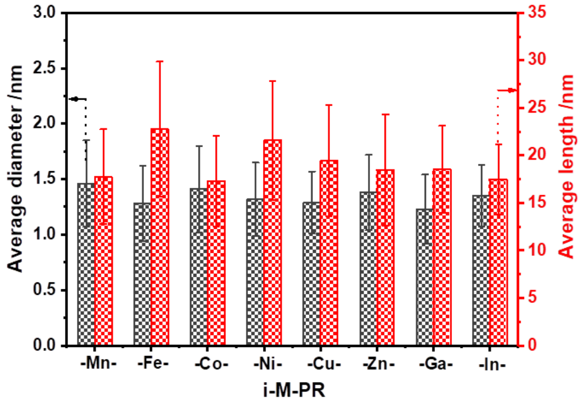

Supplementary Figure 13. Size statistics. Summary of distributions of diameters and lengths for i-M-PR.

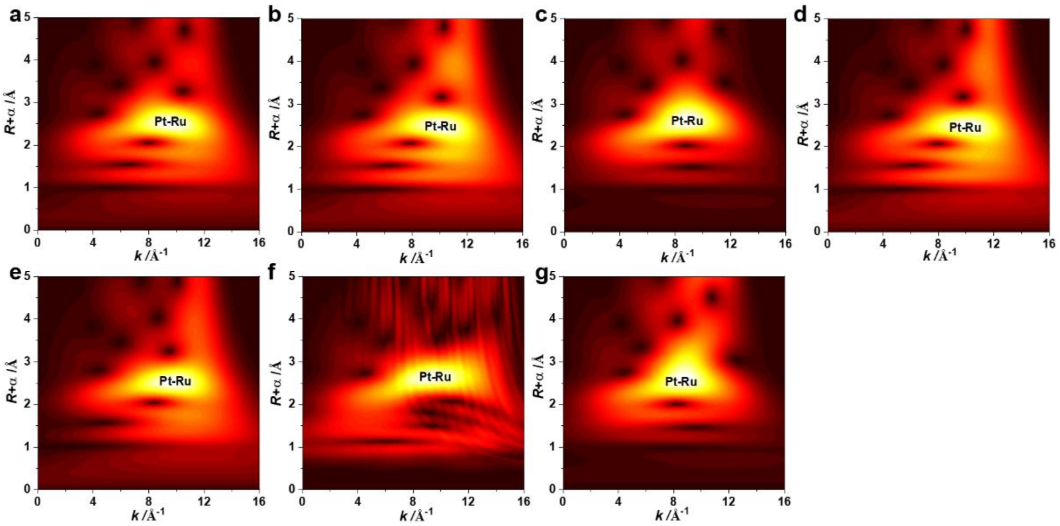

Supplementary Figure 14. Wavelet transformed. Wavelet transformed of (a) i-Mn-PR, (b) i-Fe-PR, (c) i-Co-PR, (d) i-Ni-PR, (e) i-Cu-PR, (f) i-Zn-PR and (g) i-Ga-PR at Pt L_3-edge.

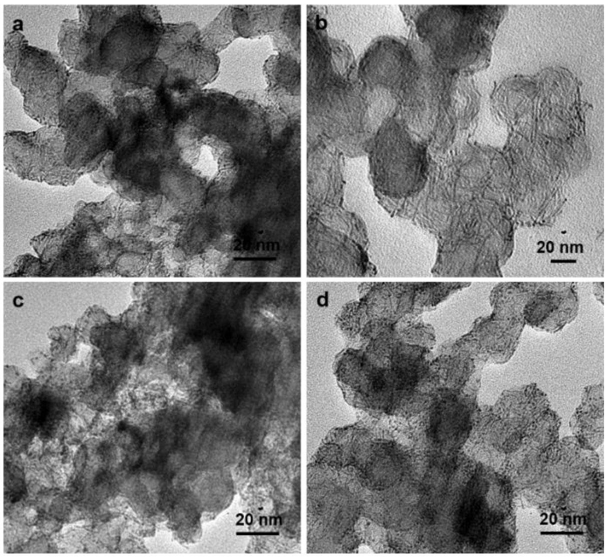

Supplementary Figure 15. Morphology characterization. TEM images of (a) i-In_2-PR/C, (b) i-$In_{3.9}$-PR/C, (c) i-$In_{5.1}$-PR/C, and (d) i-$In_{6.3}$-PR/C.

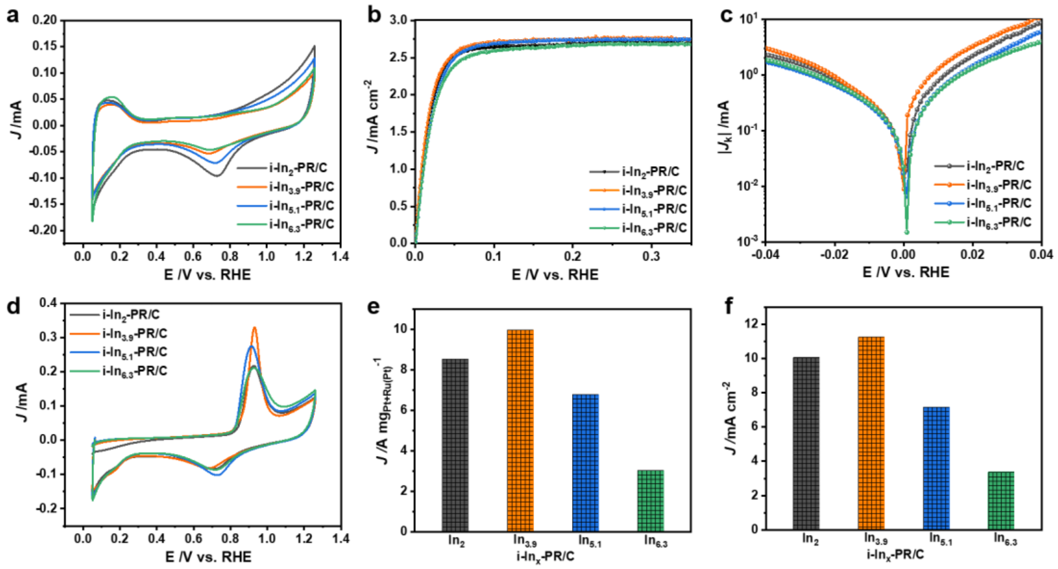

Supplementary Figure 16. HOR activity. HOR performance over i-In-PR/C with different contents of In. (a) CV curves in 0.1 M $HClO_4$ with a sweep rate of 50 mV/s, (b) HOR polarization curves in H_2 saturated aqueous solution of 0.1 M KOH with a sweep rate of 50 mV s^{-1} and a rotation rate of 1600 rpm, (c) Tafel plots, (d) CO-stripping curves, (e) normalized mass activity and (f) specific activity at an overpotential of 50 mV vs. RHE of different i-In$_x$-PR/C.

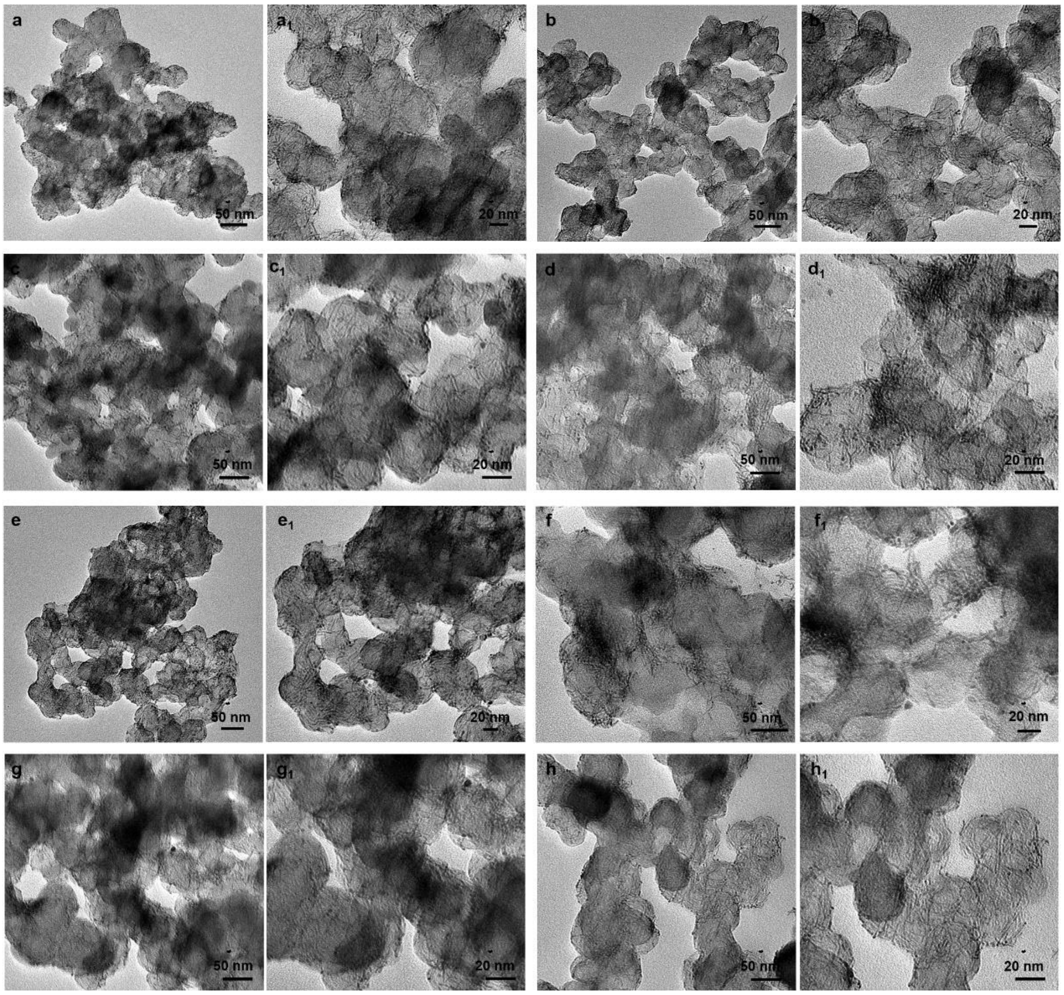

Supplementary Figure 17. Morphology characterization. TEM images of (a, a_1) i-Mn-PR/C, (b, b_1) i-Fe-PR/C, (c, c_1) i-Co-PR/C, (d, d_1) i-Ni-PR/C, (e, e_1) i-Cu-PR/C, (f, f_1) i-Zn-PR/C, (g, g_1) i-Ga-PR/C, and (h, h_1) i-In-PR/C.

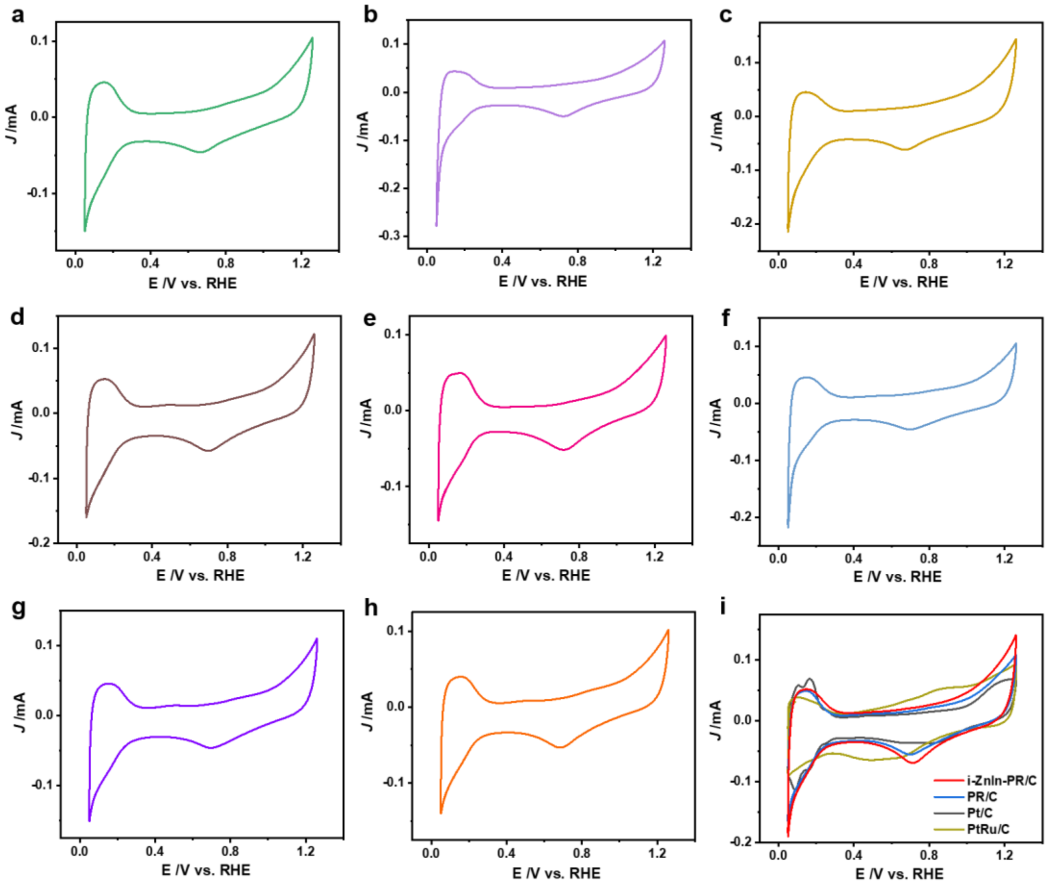

Supplementary Figure 18. CV measurements. CV curves of (a) i-Mn-PR/C, (b) i-Fe-PR/C, (c) i-Co-PR/C, (d) i-Ni-PR/C, (e) i-Cu-PR/C, (f) i-Zn-PR/C, (g) i-Ga-PR/C, (h) i-In-PR/C, and (i) commercial Pt/C, commercial PtRu/C, PR/C and i-ZnIn-PR/C recorded in 0.1 M $HClO_4$ solution with a sweep rate of 50 mV s^{-1}.

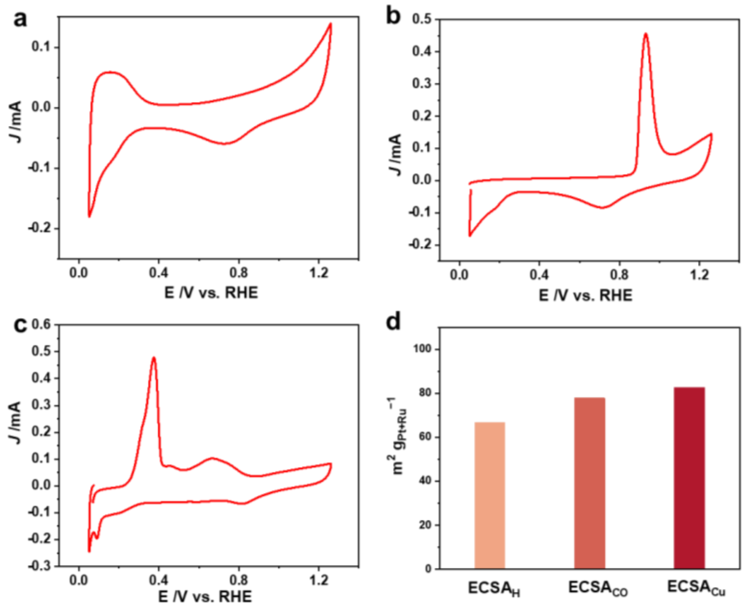

Supplementary Figure 19. ECSA measurements. (a) Hydrogen adsorption/desorption for measuring the surface area of i-ZnIn-PR/C at 50 mV s^{-1} in 0.1 M $HClO_4$. (b) CO stripping quantifies the surface area of i-ZnIn-PR/C at 50 mV s^{-1} in 0.1 M $HClO_4$. (c) Underpotential deposition of Cu for measuring the surface area of i-ZnIn-PR/C at 50 mV s^{-1} in 0.5 M $HClO_4$ in the presence of 5 mM $CuSO_4$. (d) ECSAs of i-M-PR/C at 50 mV (vs. RHE).

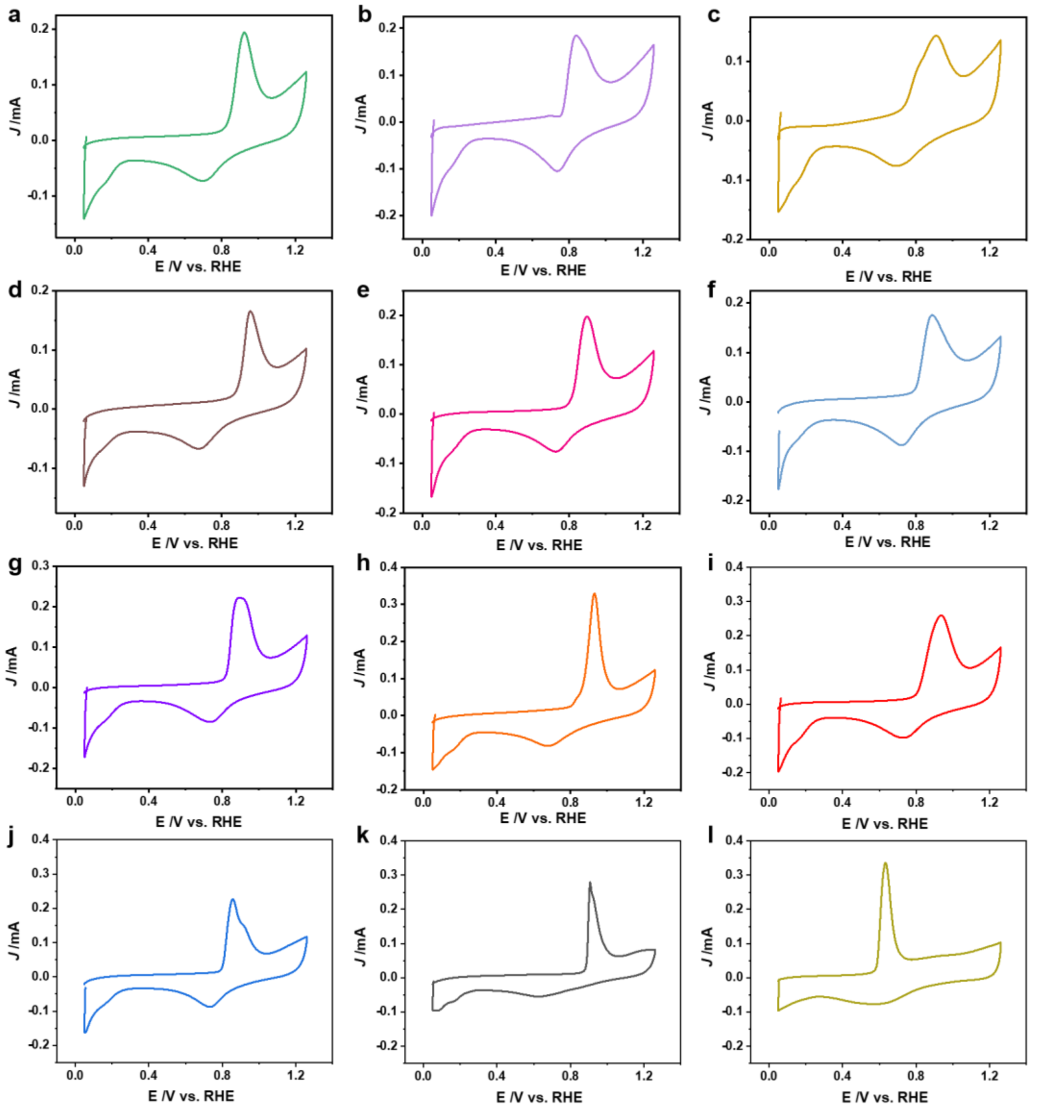

Supplementary Figure 20. CO-stripping curves. CO-stripping curves of (a) i-Mn-PR/C, (b) i-Fe-PR/C, (c) i-Co-PR/C, (d) i-Ni-PR/C, (e) i-Cu-PR/C, (f) i-Zn-PR/C, (g) i-Ga-PR/C, (h) i-In-PR/C, (i) i-ZnIn-PR/C (j) PR/C, (k) commercial Pt/C, and (l) commercial PtRu/C recorded in 0.1 M $HClO_4$ solution at a scan rate of 50 mV s^{-1}.

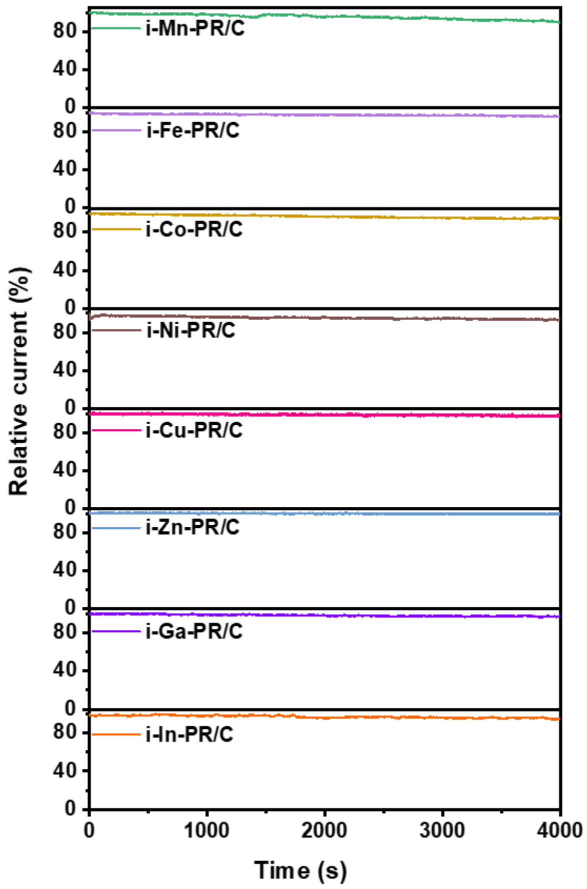

Supplementary Figure 21. Stability test. Relative current-time chronoamperometry of different i-M-PR/C in H_2-saturated 0.1 M KOH solution at an overpotential of 100 mV vs. RHE.

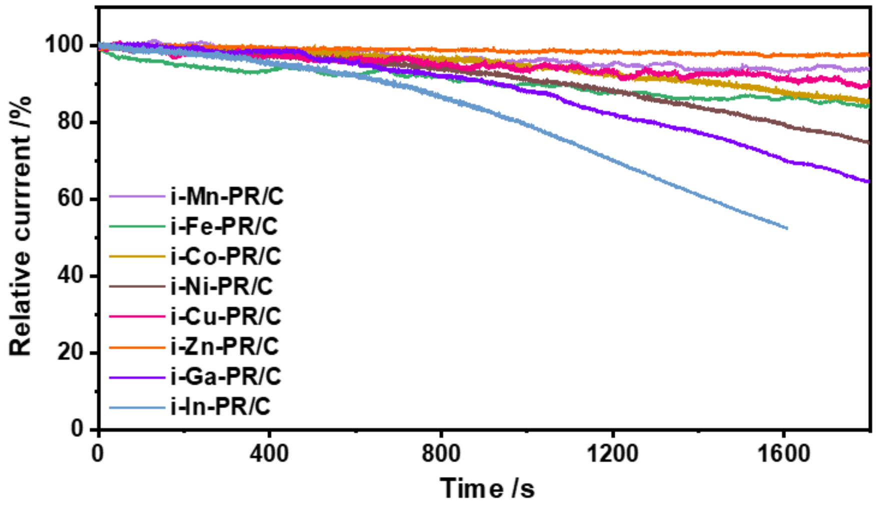

Supplementary Figure 22. CO-poisoning. Relative current-time chronoamperometry of different i-M-PR/C in 1000 ppm CO 0.1 M KOH solution at an overpotential of 100 mV vs. RHE.

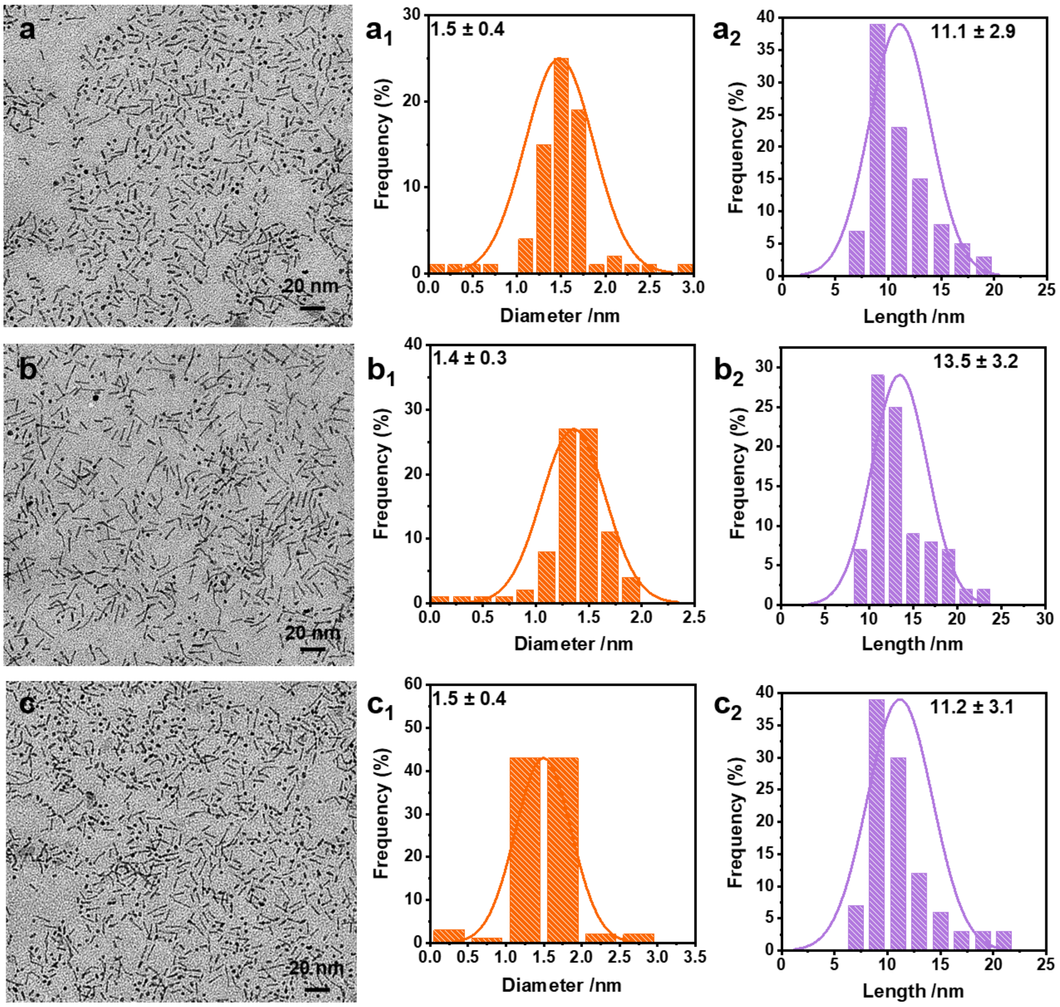

Supplementary Figure 23. TEM images and size distributions. TEM images of (a) i-$Zn_1In_{2.2}$-PR, (b) i-$Zn_1In_{1.1}$-PR, and (c) i-$Zn_{1.8}In_1$-PR. (a_1, b_1, c_1) Diameter and (a_2, b_2, c_2) length distribution of (a_1, a_2) i-$Zn_1In_{2.2}$-PR, (b_1, b_2) i-$Zn_1In_{1.1}$-PR, and (c_1, c_2) i-$Zn_{1.8}In_1$-PR.

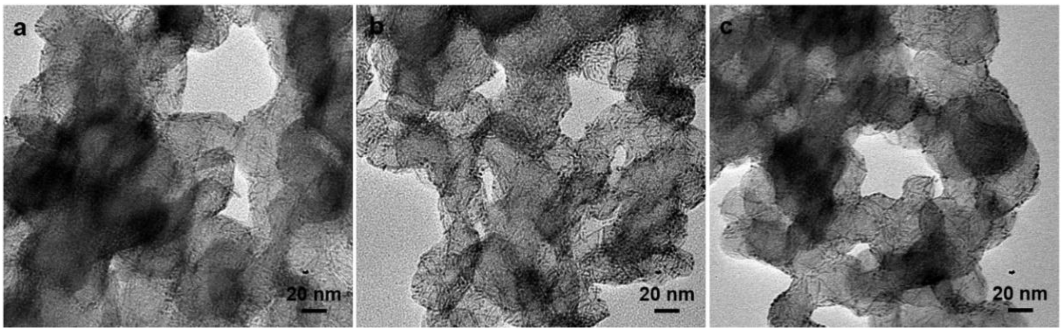

Supplementary Figure 24. Morphology characterization. TEM images of (a) i-$Zn_1In_{2.2}$-PR/C, (b) i-$Zn_1In_{1.1}$-PR/C, and (c) i-$Zn_{1.8}In_1$-PR/C.

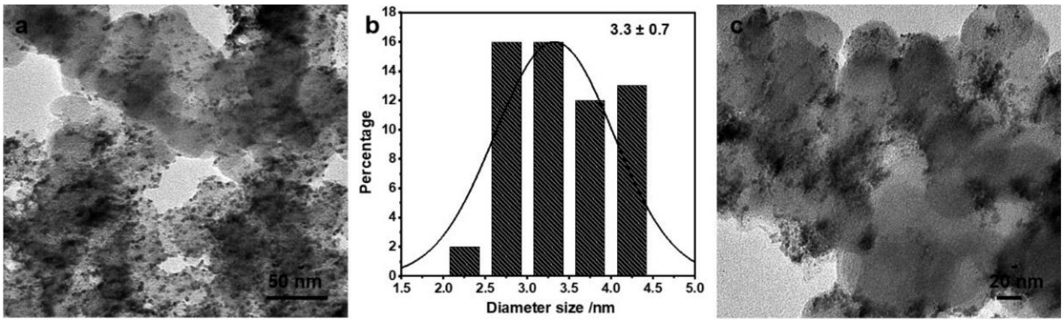

Supplementary Figure 25. TEM images and size distributions. (a) TEM image and (b) size distribution of commercial Pt/C. (c) TEM image of commercial PtRu/C.

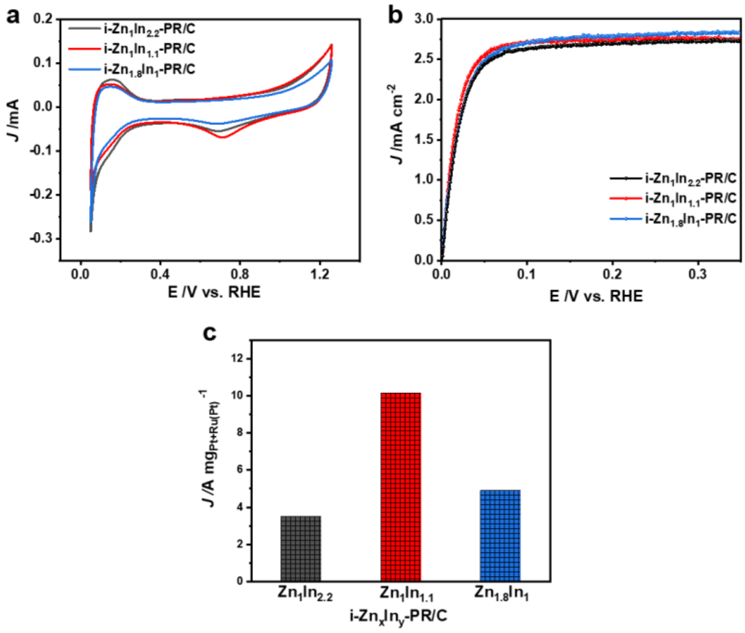

Supplementary Figure 26. CV and HOR activity. (a) CV curves recorded in 0.1 M $HClO_4$ solution with a sweep rate of 50 mV s^{-1}, (b) HOR polarization curves recorded in H_2-saturated 0.1 M KOH with a sweeping rate of 50 mV s^{-1} at a rotation rate of 1600 rpm, and (c) the electrochemically mass activity at an overpotential of 50 mV (vs. RHE) for i-$Zn_1In_{2.2}$-PR/C, i-$Zn_1In_{1.1}$-PR/C, i-$Zn_{1.8}In_1$-PR/C.

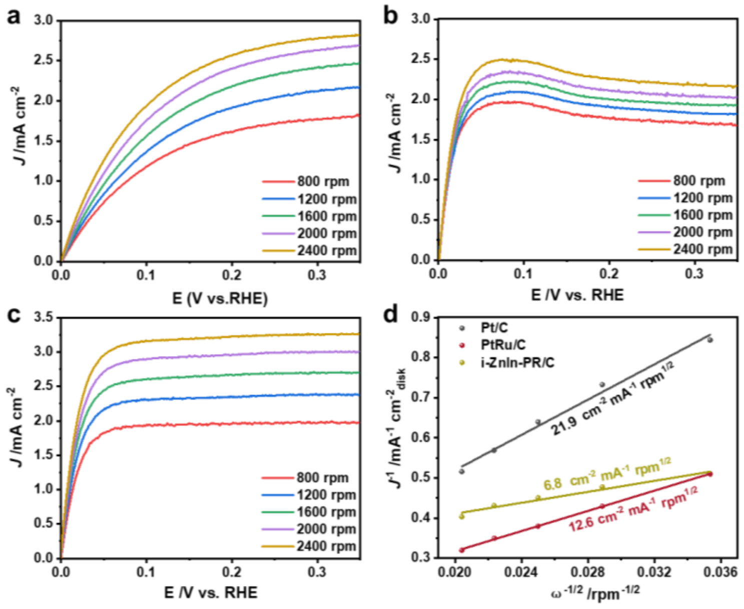

Supplementary Figure 27. HOR activity. Polarization curves of (a) commercial Pt/C, (b) commercial PtRu/C, and (c) i-ZnIn-PR/C at different rotation speeds, respectively. (d) Koutecky–Levich plots of commercial Pt/C, commercial PtRu/C, and i-ZnIn-PR/C at an overpotential of 100 mV.

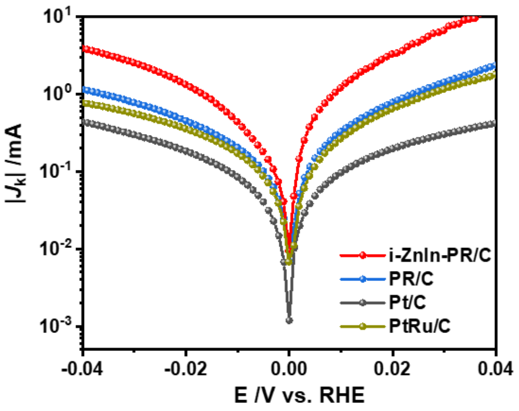

Supplementary Figure 28. Tafel plots. Representative HOR Tafel plots of kinetic current density of commercial Pt/C, PtRu/C, PR/C, and i-ZnIn-PR/C.

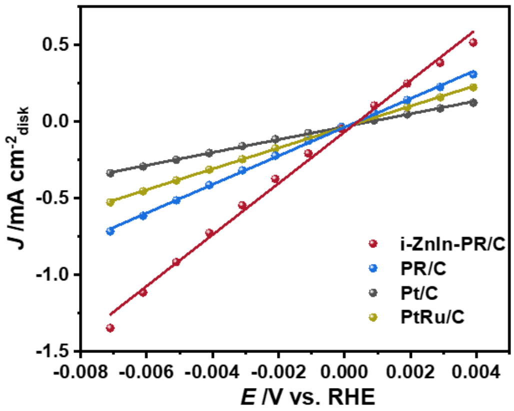

Supplementary Figure 29. Micro-polarization curves. Fitting the micro-polarization regions of HOR of Pt/C, PtRu/C, PR/C, and i-ZnIn-PR/C.

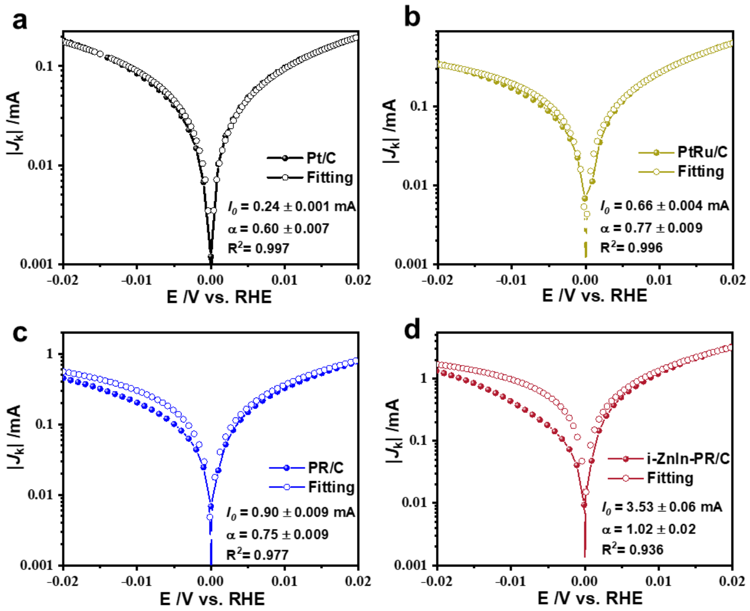

Supplementary Figure 30. Butler-Volmer fitting curves. Butler-Volmer fitting on the HER/HOR polarization curves for (a) Pt/C, (b) PtRu/C, (c) PR/C, (d) i-ZnIn-PR/C.

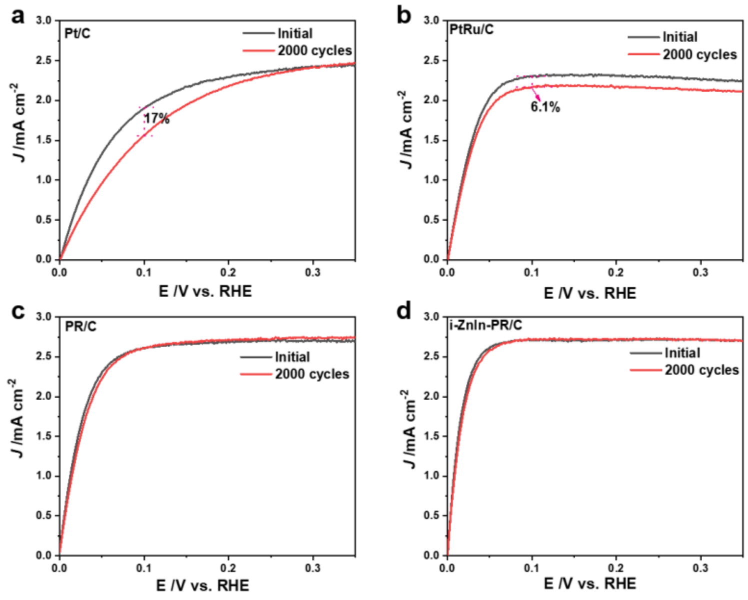

Supplementary Figure 31. HOR polarization curves. Polarization curves of (a) commercial Pt/C, (b) commercial PtRu/C, (c) PR/C, and (d) i-ZnIn-PR/C before and after 2000 cycles of ADT between −0.1 and 0.4 V vs. RHE.

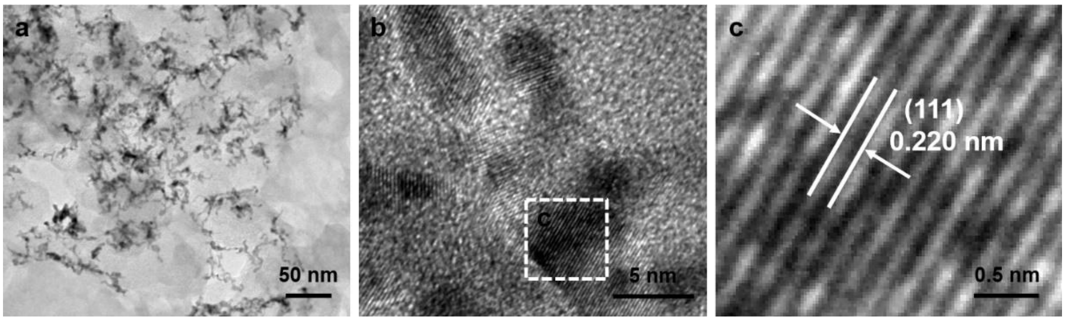

Supplementary Figure 32. Morphology characterization. (a) TEM image, (b, c) HRTEM images of i-ZnIn-PR/C after 2000 cycles of ADT.

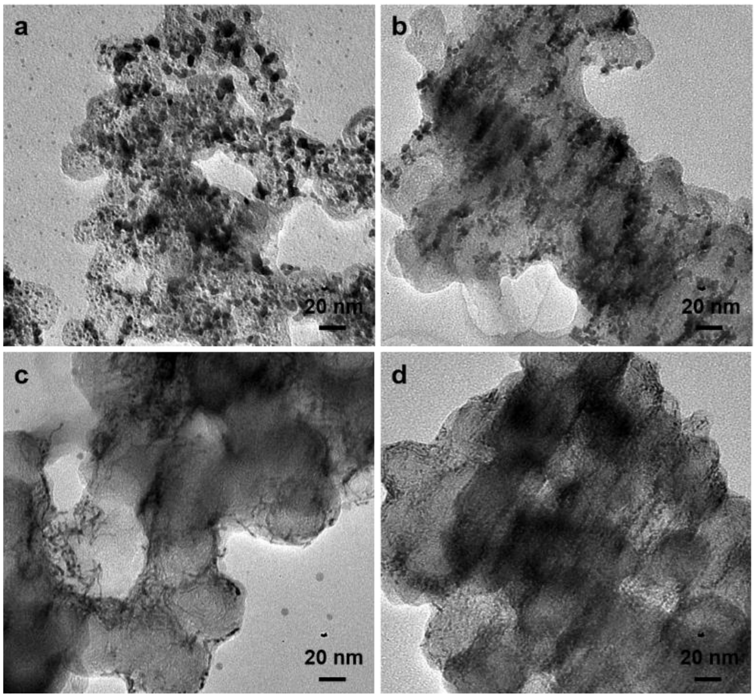

Supplementary Figure 33. Morphology characterization. TEM images of the spent (a) commercial Pt/C, (b) commercial PtRu/C, (c) PR/C, and (d) i-ZnIn-PR/C after 2000 cycles of ADT.

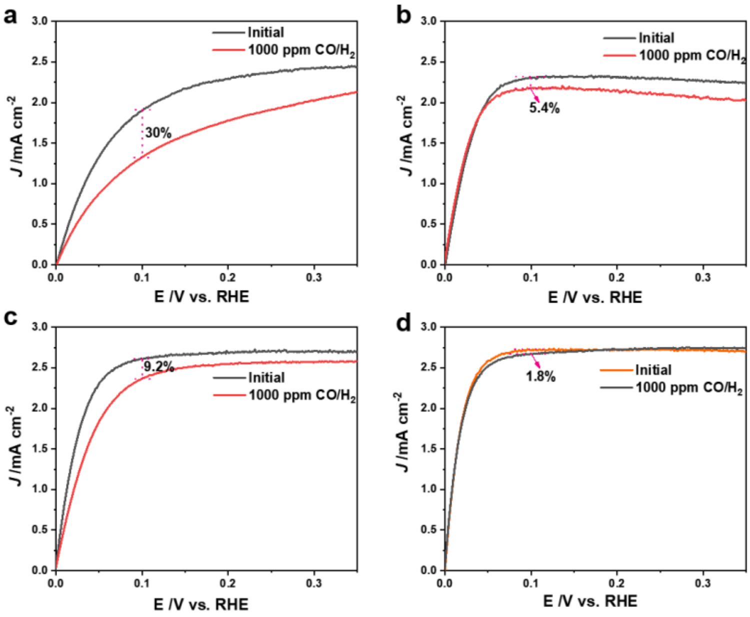

Supplementary Figure 34. HOR polarization curves. Polarization curves of (a) commercial Pt/C, (b) commercial PtRu/C, (c) PR/C, and (d) i-ZnIn-PR/C in 1000 ppm CO/H_2-saturated 0.1 M KOH.

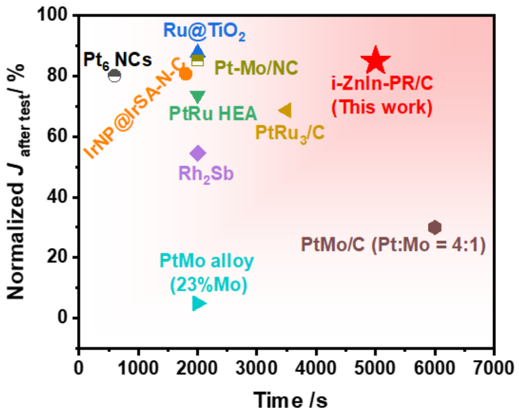

Supplementary Figure 35. Comparison of resistance CO-poisoning. Comparison of resistance to CO-poisoning between i-ZnIn-PR/C and other reported catalysts. Normalized $J_{\text{after test}}$ is the ratio of reserved current to the initial value after stability test.

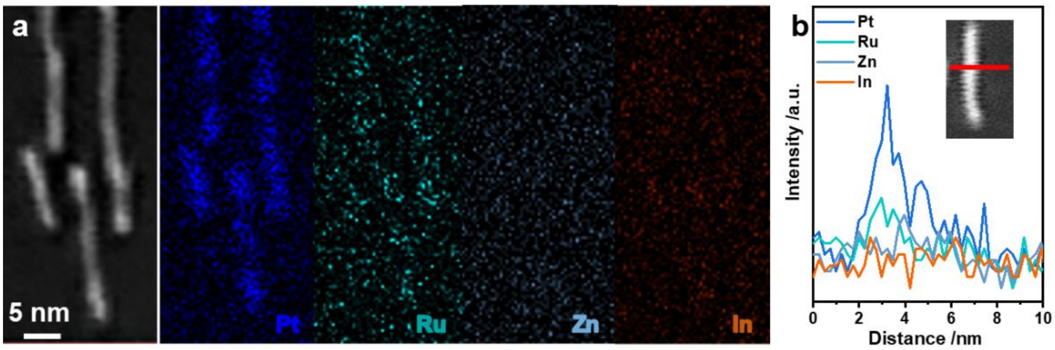

Supplementary Figure 36. TEM characterization. (a) HAADF-STEM image with elemental mappings and (b) EDS line-scanning elemental profile of i-ZnIn-PR.

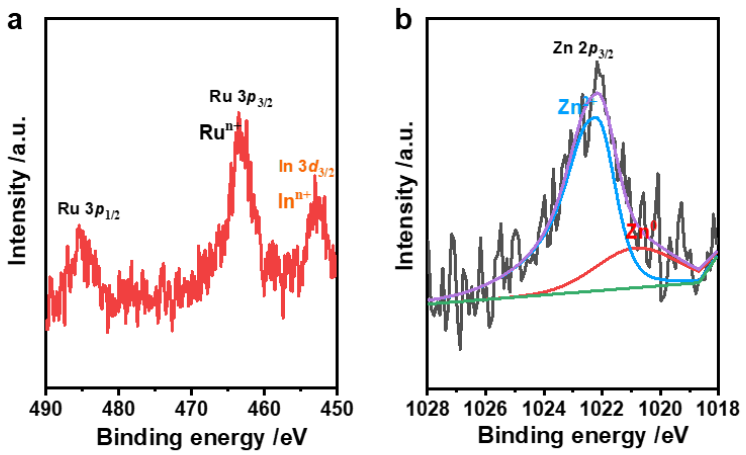

Supplementary Figure 37. XPS characterization. (a) Ru 3*p* XPS and (b) Zn 2*p* XPS spectra of i-ZnIn-PR.

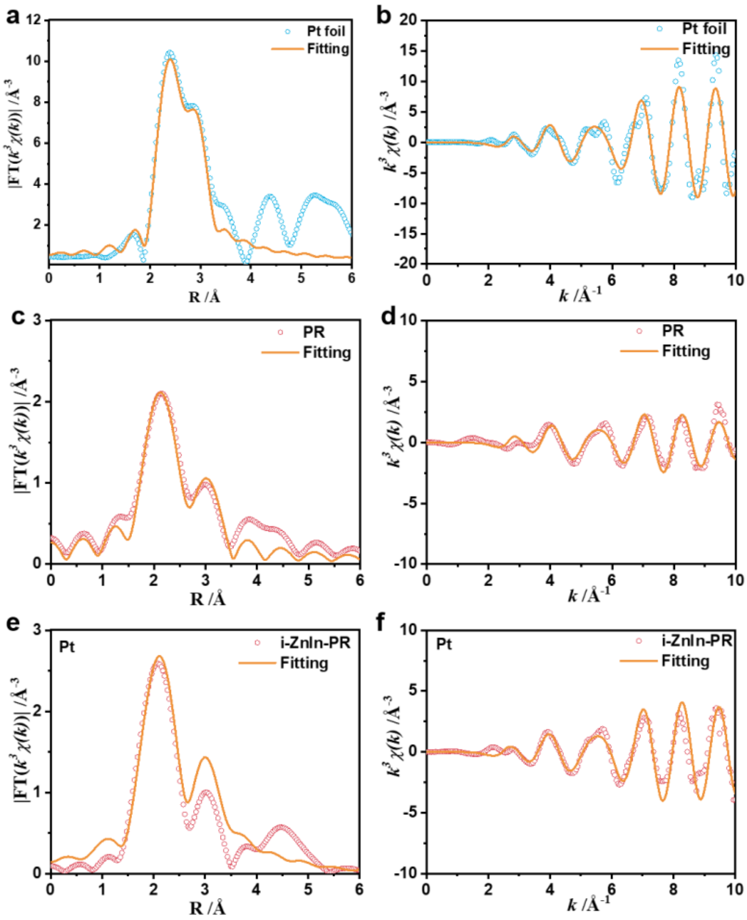

Supplementary Figure 38. EXAFS curves and corresponding fitting results. (a, c, e) *R*-space and (b, d, f) *k*-space fitting curves results of Pt L_3-edge for (a, b) Pt foil, (c, d) PR and (e, f) i-ZnIn-PR.

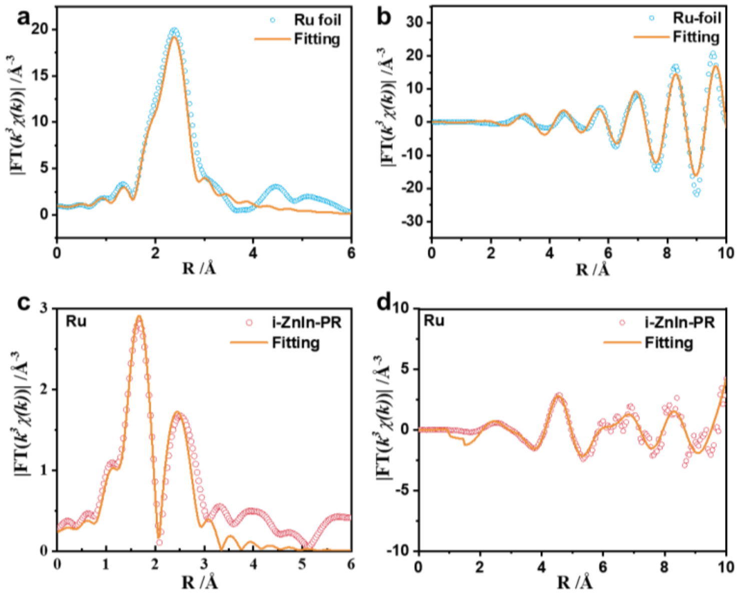

Supplementary Figure 39. EXAFS curves and corresponding fitting results. (a, c) *R*-space and (b, d) *k*-space fitting curves results of Ru *K*-edge for (a, b) Ru foil and (c, d) i-ZnIn-PR.

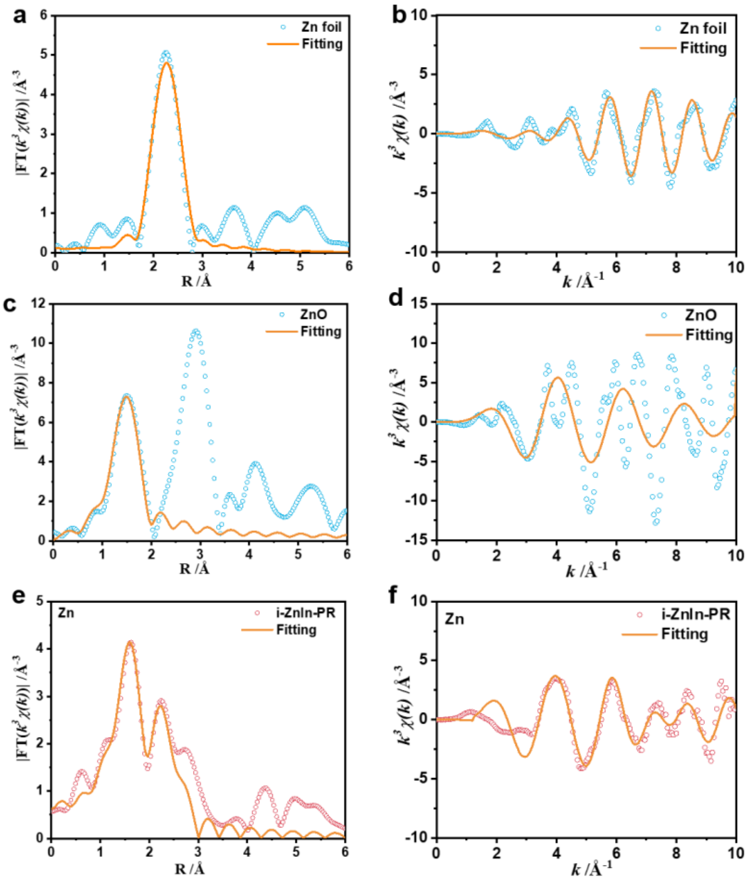

Supplementary Figure 40. EXAFS curves and corresponding fitting results. (a, c, e) *R*-space and (b, d, f) *k*-space fitting curves results of (a, b) Zn foil, (c, d) ZnO, and (e, f) i-ZnIn-PR.

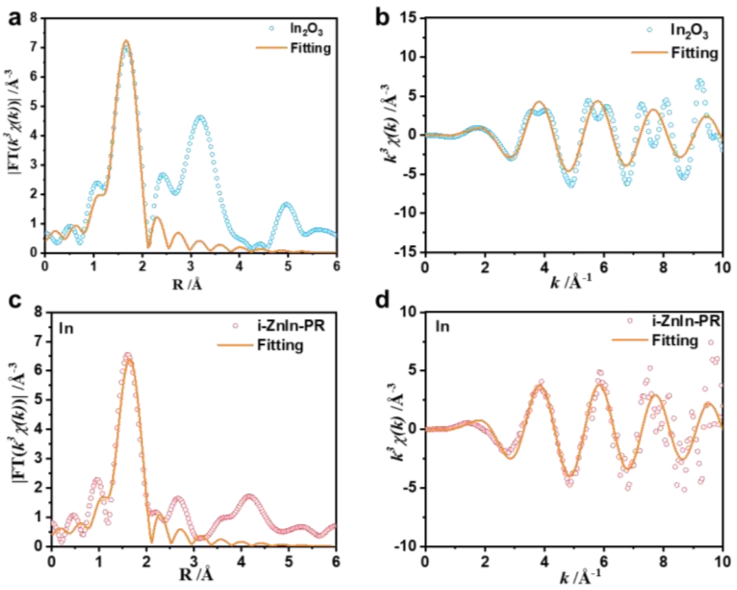

Supplementary Figure 41. EXAFS curves and corresponding fitting results. (a, c) *R*-space and (b, d) *k*-space fitting curves results of (a, b) In_2O_3 and (c, d) i-ZnIn-PR.

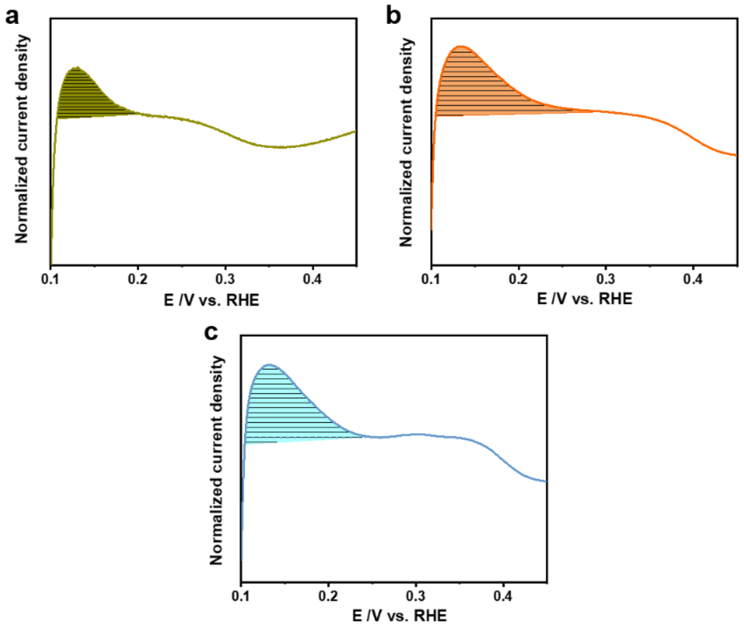

Supplementary Figure 42. CV curves. Integrated areas of weak H_2 adsorption of (a) commercial PtRu/C, (b) i-In-PR/C, and (c) i-Zn-PR/C CV curves in N_2-saturation KOH solution.

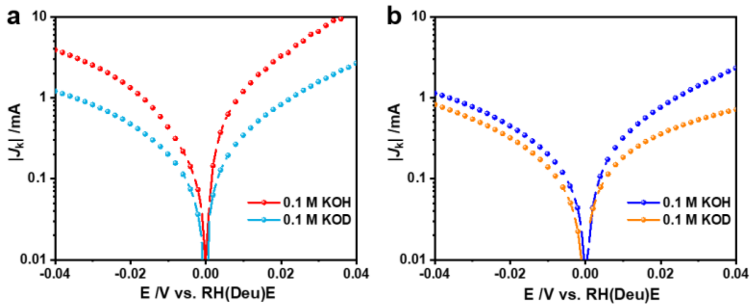

Supplementary Figure 43. Tafel plots. HOR Tafel plots of kinetic current density of (a) i-ZnIn-PR/C and (b) PR/C derived from Fig. 5d RHE or RDeuH.

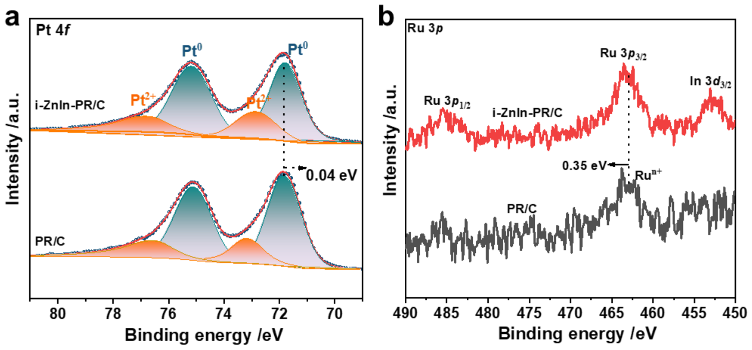

Supplementary Figure 44. XPS characterization. (a) Pt 4*f* and (b) Ru 3*p* XPS spectra of PR/C and i-ZnIn-PR/C.

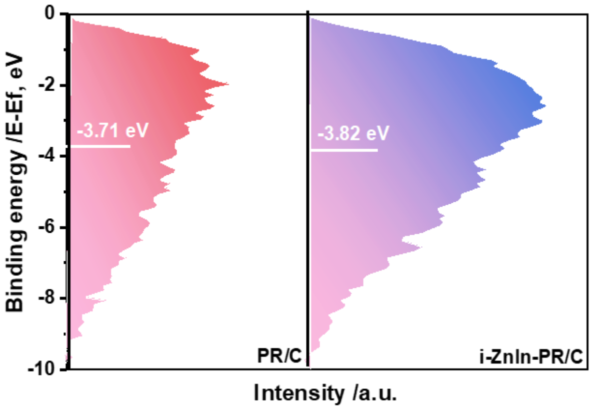

Supplementary Figure 45. XPS characterization. *d*-band center of PR/C and i-ZnIn-PR/C.

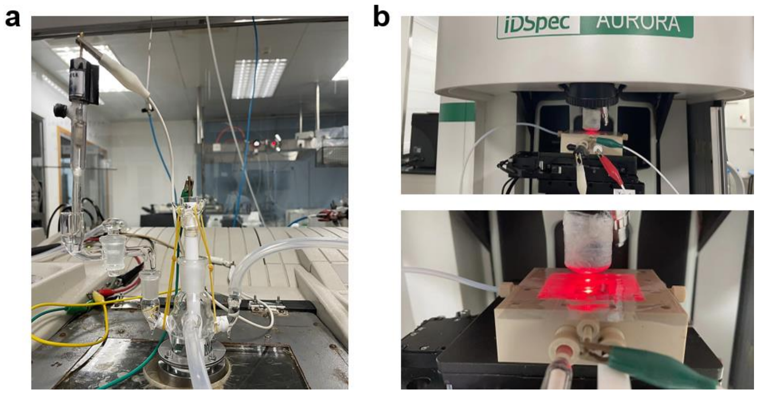

Supplementary Figure 46. In situ testing device. (a) In situ DRIFTS electrochemical cell, (b) In situ Raman electrochemical cell.

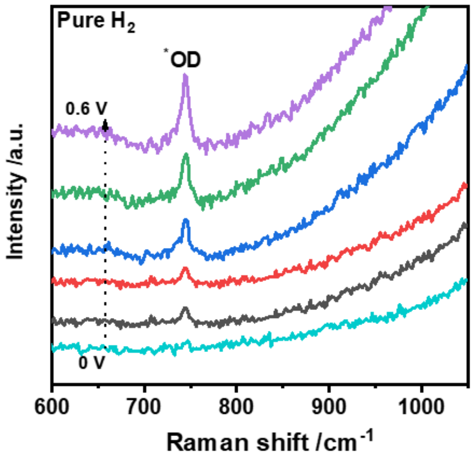

Supplementary Figure 47. Raman characterization. In situ Raman spectra of i-ZnIn-PR/C collected in H_2-saturated KOD solution (0.1 M).

Supplementary Table 1. Compositions of i-M-PR/C catalysts.

Catalyst	Composition/molar%		
	**Pt**	**Ru**	**M**
i-In_2-PR/C	71.1	26.9	2.0
i-$In_{3.6}$-PR/C	72.8	23.3	3.9
i-$In_{5.1}$-PR/C	70.1	24.8	5.1
i-$In_{6.3}$-PR/C	66.8	26.9	6.3
PR/C	77.4	22.6	−
i-Mn-PR/C	73.2	22.8	4.0
i-Fe-PR/C	73.2	22.4	4.4
i-Co-PR/C	72.7	22.6	4.7
i-Ni-PR/C	71.8	23.7	4.5
i-Cu-PR/C	73.4	22.2	4.4
i-Zn-PR/C	71.7	24.1	4.2
i-Ga-PR/C	73.9	22.2	3.9

Supplementary Table 2. The amounts of noble metal of i-M-PR/C.

Catalyst	Metal contents/wt%	
	Pt	Ru
PR/C	18.4	2.8
i-Mn-PR/C	21.7	3.5
i-Fe-PR/C	19.6	3.1
i-Co-PR/C	22.0	3.5
i-Ni-PR/C	17.1	2.9
i-Cu-PR/C	16.7	2.6
i-Zn-PR/C	17.2	3.0
i-Ga-PR/C	18.7	2.9
i-In_2-PR/C	19.9	3.9
i-$In_{3.9}$-PR/C	23.2	3.9
i-$In_{5.1}$-PR/C	17.0	3.1
i-$In_{6.3}$-PR/C	19.5	4.1
i-$Zn_1In_{2.2}$-PR/C	21.7	3.4
i-$Zn_1In_{1.1}$-PR/C	18.2	2.7
i-$Zn_{1.8}In_1$-PR/C	17.7	2.9

Supplementary Table 3. The loading amounts noble metals and ECSAs of i-M-PR/C.

**Catalyst**	**Loading (μg_{Pt} cm^{-2})**	**Loading (μg_{Pt+Ru} cm^{-2})**	**ECSA (m^2 g_{Pt}^{-1})**	**ECSA (m^2 g_{Pt+Ru}^{-1})**
Pt/C	10.2	10.2	55.9	55.9
PtRu/C	3.4	10.2	206.1	69.1
PR/C	10.2	11.7	57.6	64.2
i-Mn-PR/C	10.2	11.8	74.8	64.5
i-Fe-PR/C	10.2	12.1	82.9	70.2
i-Co-PR/C	10.2	11.8	82.1	70.8
i-Ni-PR/C	10.2	11.8	60.0	51.3
i-Cu-PR/C	10.2	11.8	70.7	61.0
i-Zn-PR/C	10.2	12.0	80.0	68.1
i-Ga-PR/C	10.2	11.8	85.2	73.8
i-In_2-PR/C	10.2	12.2	71.9	60.2
i-$In_{3.9}$-PR/C	10.2	11.9	81.2	69.7
i-$In_{5.1}$-PR/C	10.2	12.1	87.6	73.9
i-$In_{6.3}$-PR/C	10.2	12.3	87.2	72.3
i-ZnIn-PR/C	10.2	11.7	89.5	77.8

Supplementary Table 4. The specific activities, mass activities, and exchange current for i-M-PR/C.

Catalyst	$J_{m,\ 50mV}$ (A mg_{Pt}^{-1})	$J_{m,\ 50mV}$ (A mg_{Pt+Ru}^{-1})	$J_{s,\ 50mV}$ (mA cm^{-2})	I_0 (mA)	α
Pt/C	0.27	0.27	0.48	0.24	0.60
PtRu/C	3.7	1.24	1.80	0.66	0.77
PR/C	1.84	1.60	2.50	0.90	0.75
i-Mn-PR/C	6.89	5.94	8.75	1.68	0.95
i-Fe-PR/C	6.77	5.74	8.17	1.74	0.99
i-Co-PR/C	6.98	6.02	8.50	2.08	0.95
i-Ni-PR/C	1.98	1.69	3.29	0.79	0.78
i-Cu-PR/C	2.33	2.01	3.30	0.85	0.82
i-Zn-PR/C	5.37	4.57	6.72	2.09	0.87
i-Ga-PR/C	3.32	2.87	3.89	1.64	0.81
i-In_2-PR/C	8.53	7.14	11.86	2.28	1.01
i-$In_{3.9}$-PR/C	9.96	8.51	12.21	3.01	0.99
i-$In_{5.1}$-PR/C	6.77	5.72	7.74	1.46	0.99
i-$In_{6.3}$-PR/C	3.03	2.50	3.46	1.38	0.79
i-ZnIn-PR/C	11.7	10.2	13.11	3.53	1.02

Supplementary Table 5. Compositions of i-Zn_xIn_y-PR/C catalysts.

Catalyst	Compositions/mol%			
	**Pt**	**Ru**	**Zn**	**In**
i-$Zn_1In_{2.2}$-PR/C	72.6	22.0	1.7	3.8
i-$Zn_1In_{1.1}$-PR/C	70.4	25.4	1.9	2.2
i-$Zn_{1.8}In_1$-PR/C	72.0	22.8	2.1	1.2

Supplementary Table 6. Summary of the reported catalysts for HOR.

**Catalyst**	**Loading (μg$_{NM}$ cm^{-2})**	***J*$_m$ (A mg$_{NM}^{-1}$)**	***J*$_s$ (mA cm^{-2})**	**Ref.**
$IrWO_x/C$	2.01	2.16@50 mV	1.56	1
$Pt_{0.1}Ru_{0.9}$	8	1.9@50 mV	6.5	2
PtRu NWs	20	0.6@50 mV	2.2	3
$PtRu/Mo_2C$-TaC	13	0.403@50 mV	0.28	4
Ru_7Ni_3/C	3.9	9.4@50 mV	23.4	5
$RuNi_1$	8.8	2.7@50 mV	/	6
$Ru@TiO_2$	230	0.29@50 mV	/	7
IrNi@Ir/C	10	1.12@50 mV	/	8
PdCu/C-500 °C	12.5	1.727@50 mV	2.922	9
0.38 CeO_x-Pd/C	13	/	0.118	10
IrNi@PdIr/C	19.7	0.854@50 mV	1.656	11
HEA SNWs/C	8.8	6.75@50 mV	8.96	12
Ir_9Ru_1/C	3.5	0.34@10 mV	0.9	13
Ir ONA/C	30	0.135@10 mV	0.518	14
**i-ZnIn-PR/C**	**11.7**	**10.2@50 mV**	**13.11**	**This work**

NM* represents noble metal.

Supplementary Table 7. Comparison of CO tolerance between i-ZnIn-PR/C and other reported catalysts.

Catalyst	Potential (V)	Normalized[a] $J_{after\ test}$	Time (s)	CO concentration	Ref.
IrNP@IrSA-N-C	0.1	19.4%	2000	1000 ppm	15
Ru@TiO_2	0.1	12.4%	2000	1000 ppm	7
HEA SNWs	0.1	26.4%	2000	1000 ppm	12
Rh_2Sb	0.1	45.5%	2000	1000 ppm	16
$PtRu_3$/C	0.4	31.4%	3500	1000 ppm	17
PtMo alloy (23%Mo)	0.1	95%	2000	1000 ppm	18
PtMo/C (Pt:Mo 4:1)	0.1	70%	6000	1000 ppm	18
Pt-Mo/NC	0.1	15%	2000	100 ppm	19
Pt_6 NCs	0.1	20%	600	100 ppm	20
**i-ZnIn-PR/C**	**0.1**	**15.1%**	**5000**	**1000 ppm**	**This work**

[a]: The normalized $J_{after\ test}$ represents the ratio of the residual current to the initial current.

Supplementary Table 8. Structural parameters of Pt foil and i-ZnIn-PR extracted from the EXAFS fitting.

Sample	Shell	CN	R (Å)	σ^2/Å^2	R-factor
Pt foil	Pt−Pt	12	2.8 ± 0.01	0.0050 ± 0.0011	0.013
PR	Pt−Pt (Ru)	3.99 ± 2.5	2.7 ± 0.03	0.015 ± 0.003	0.018
i-ZnIn-PR	Pt−Pt (M)	6.5 ± 1.4	2.7 ± 0.02	0.0066	0.019

Note: CN is the coordination number; R is interatomic distance; σ^2 is the Debye-Waller factor (a measure of thermal and static disorder in absorber-scatterer distances); S_0^2 for Pt-Pt was set as 0.84, which is obtained from the experimental EXAFS fit of the Pt foil reference by fixing CN to the known crystallographic value and was fixed to all samples; R-factor is used to value the goodness of the fitting.

Supplementary Table 9. Structural parameters of Ru foil and i-ZnIn-PR extracted from the EXAFS fitting.

Sample	Shell	CN	R (Å)	σ^2/Å^2	R-factor
Ru foil	Ru−Ru	12	2.7 ± 0.01	0.0026 ± 0.0013	0.021
i-ZnIn-PR	Ru−O	3.8 ± 1.43	2.1 ± 0.02	0.0100 ± 0.0036	0.016
i-ZnIn-PR	Ru−Ru (M)	5.2 ± 1.4	2.7 ± 0.03	0.0181 ± 0.0040	0.016

Note: CN is the coordination number; R is interatomic distance; σ^2 is the Debye-Waller factor (a measure of thermal and static disorder in absorber-scatterer distances); S_0^2 for Ru-Ru was set as 0.64, which is obtained from the experimental EXAFS fit of the Ru foil reference by fixing CN to the known crystallographic value and was fixed to all samples; R-factor is used to value the goodness of the fitting.

Supplementary Table 10. Structural parameters of Zn foil, ZnO and i-ZnIn-PR extracted from the EXAFS fitting.

Sample	Shell	CN	R (Å)	σ^2/Å^2	R-factor
Zn foil	Zn−Zn	6	2.6 ± 0.02	0.0130 ± 0.0011	0.019
ZnO	Zn−O	4	2.0 ± 0.03	0.0093 ± 0.0024	0.017
i-ZnIn-PR	Zn−O	3.02 ± 0.22	2.06 ± 0.03	0.0125	0.019
i-ZnIn-PR	Zn−Pt	1.61 ± 0.37	2.66 ± 0.04	0.0077	0.019

Note: CN is the coordination number; R is interatomic distance; σ^2 is the Debye-Waller factor (a measure of thermal and static disorder in absorber-scatterer distances); S_0^2 for Zn-Zn was set as 0.70, which is obtained from the experimental EXAFS fit of the Zn foil reference by fixing CN to the known crystallographic value and was fixed to all samples; R-factor is used to value the goodness of the fitting.

Supplementary Table 11. Structural parameters of In_2O_3 and i-ZnIn-PR extracted from the EXAFS fitting.

Sample	Shell	CN	R (Å)	σ^2/Å^2	R-factor
In_2O_3	In−O	6	2.17 ± 0.01	0.0059 ± 0.0016	0.004
i-ZnIn-PR	In−O	5.21 ± 1.26	2.16 ± 0.03	0.0060 ± 0.0036	0.019
i-ZnIn-PR	In−Pt	0.54 ± 0.04	3.23 ± 0.13	0.0021 ± 0.0037	0.019

Note: CN is the coordination number; R is interatomic distance; σ^2 is the Debye-Waller factor (a measure of thermal and static disorder in absorber-scatterer distances); S_0^2 for In-O was set as 0.99, which is obtained from the experimental EXAFS fit of the In_2O_3 reference by fixing CN to the known crystallographic value and was fixed to all samples; R-factor is used to value the goodness of the fitting.

References

1. Fu, L. et al. Discrepant roles of adsorbed OH^* species on $IrWO_x$ for boosting alkaline hydrogen electrocatalysis. *Sci. Bull.* **65**, 1735−1742 (2020).
2. Strmcnik, D. et al. Improving the hydrogen oxidation reaction rate by promotion of hydroxyl adsorption. *Nat. Chem.* **5**, 300−306 (2013).
3. Scofield, M. E. et al. Role of chemical composition in the enhanced catalytic activity of Pt-based alloyed ultrathin nanowires for the hydrogen oxidation reaction under alkaline conditions. *ACS Catal.* **6**, 3895−3908 (2016).
4. Hamo, E. R. et al. Carbide-supported PtRu catalysts for hydrogen oxidation reaction in alkaline electrolyte. *ACS Catal.* **11**, 932−947 (2021).
5. Xue, Y. et al. A highly-active, stable and low-cost platinum-free anode catalyst based on RuNi for hydroxide exchange membrane fuel cells. *Nat. Commun.* **11**, 5651 (2020).
6. Mao, J. et al. Isolated Ni atoms dispersed on Ru nanosheets: High-performance electrocatalysts toward hydrogen oxidation reaction. *Nano Lett.* **20**, 3442−3448 (2020).
7. Zhou, Y. et al. Lattice-confined Ru clusters with high CO tolerance and activity for the hydrogen oxidation reaction. *Nat. Catal.* **3**, 454−462 (2020).
8. Liu, D. et al. One-pot synthesis of IrNi@Ir core-shell nanoparticles as highly active hydrogen oxidation reaction electrocatalyst in alkaline electrolyte. *Nano Energy* **59**, 26−32 (2019).
9. Qiu, Y. et al. BCC-phased PdCu alloy as a highly active electrocatalyst for hydrogen oxidation in alkaline electrolytes. *J. Am. Chem. Soc.* **140**, 16580−16588 (2018).
10. Singh, R. K. et al. Synthesis of CeO_x-decorated Pd/C catalysts by controlled surface reactions for hydrogen oxidation in anion exchange membrane Fuel Cells. *Adv. Funct. Mater.* **30**, 2002087 (2020).
11. Qin, B. et al. A novel IrNi@PdIr/C core–shell electrocatalyst with enhanced activity and durability for the hydrogen oxidation reaction in alkaline anion exchange membrane fuel cells. *Nanoscale* **10**, 4872−4881 (2018).
12. Zhan, C. et al. Subnanometer high-entropy alloy nanowires enable remarkable hydrogen oxidation catalysis. *Nat. Commun.* **12**, 6261 (2021).
13. Wang, H. & Abruña, H. D. IrPdRu/C as H_2 oxidation catalysts for alkaline Fuel Cells. *J. Am. Chem. Soc.* **139**, 6807−6810 (2017).
14. Yang, F., Fu, L., Cheng, G., Chen, S. & Luo, W. Ir-oriented nanocrystalline assemblies with high activity for hydrogen oxidation/evolution reactions in an alkaline electrolyte. *J. Mater. Chem. A* **5**, 22959−22963 (2017).
15. Yang, X. et al. CO-tolerant PEMFC anodes enabled by synergistic catalysis between iridium single-atom aites and nanoparticles. *Angew. Chem. Int. Ed.* **60**, 26177−26183 (2021).

16. Zhang, Y. et al. Atomically isolated Rh sites within highly branched Rh_2Sb nanostructures enhance bifunctional hydrogen electrocatalysis. *Adv. Mater.* **33**, 2105049 (2021).
17. Zhang, J. et al. Engineering the near-surface of $PtRu_3$ nanoparticles to improve hydrogen oxidation activity in alkaline electrolyte. *Small* **17**, 2006698 (2021).
18. Grgur, B. N., Markovic, N. M. & Ross, P. N. The electro-oxidation of H_2 and H_2/CO mixtures on carbon-supported Pt_xMo_y alloy catalysts. *J. Electrochem. Soc.* **146**, 1613−1619 (1999).
19. Ma, M. et al. Single-atom molybdenum engineered platinum nanocatalyst for boosted alkaline hydrogen oxidation. *Adv. Energy. Mater.* **12**, 2103336 (2022).
20. AAOHN JournalWang, X. et al. Atomic-precision Pt_6 nanoclusters for enhanced hydrogen electro-oxidation. *Nat. Commun.* **13**, 1596 (2022).
